# Detecting asymptomatic carriage of *Plasmodium falciparum* in southern Ghana: utility of molecular and serological diagnostic tools

**DOI:** 10.1186/s12936-022-04078-w

**Published:** 2022-02-19

**Authors:** Hamza B. Agbana, Eric Rogier, Aminata Lo, Zakaria Abukari, Sophie Jones, Ben Gyan, Michael Aidoo, Linda E. Amoah

**Affiliations:** 1 Immunology Department, Noguchi Memorial Institute for Medical Research (NMIMR), University of Ghana, Accra, Ghana; 2 School of Life Science and Technology, University of Electronic Science and Technology of China, Chengdu, China; 3 The Centers for Disease Control and Prevention, Center for Global Health, Division of Parasitic Diseases and Malaria, Malaria Branch, Atlanta, GA USA

**Keywords:** Malaria, Bead-based multiplex, HRP2, PET-PCR, Asymptomatic, RDT, Microscopy

## Abstract

**Background:**

Asymptomatic malaria infections can serve as potential reservoirs for malaria transmission. The density of parasites contained in these infections range from microscopic to submicroscopic densities, making the accurate detection of asymptomatic parasite carriage highly dependent on the sensitivity of the tools used for the diagnosis. This study sought to evaluate the sensitivities of a variety of molecular and serological diagnostic tools at determining the prevalence of asymptomatic *Plasmodium falciparum* parasite infections in two communities with varying malaria parasite prevalence.

**Methods:**

Whole blood was collected from 194 afebrile participants aged between 6 and 70 years old living in a high (Obom) and a low (Asutsuare) malaria transmission setting of Ghana. Thick and thin blood smears, HRP2 based malaria rapid diagnostic test (RDT) and filter paper dried blood spots (DBS) were prepared from each blood sample. Genomic DNA was extracted from the remaining blood and used in *Plasmodium* specific photo-induced electron transfer polymerase chain reaction (PET-PCR) and Nested PCR, whilst the HRP2 antigen content of the DBS was estimated using a bead immunoassay. A comparison of malaria parasite prevalence as determined by each method was performed.

**Results:**

Parasite prevalence in the high transmission site of Obom was estimated at 71.4%, 61.9%, 60%, 37.8% and 19.1% by Nested PCR, the HRP2 bead assay, PET-PCR, HRP2-RDT and microscopy respectively. Parasite prevalence in the low transmission site of Asutsuare was estimated at 50.1%, 11.2%, 5.6%, 0% and 2.2% by Nested PCR, the HRP2 bead assay, PET-PCR, RDT and microscopy, respectively. The diagnostic performance of Nested PCR, PET-PCR and the HRP2 bead assay was similar in Obom but in Asutsuare, Nested PCR had a significantly higher sensitivity than PET-PCR and the HRP2 bead assay, which had similar sensitivity.

**Conclusions:**

Nested PCR exhibited the highest sensitivity by identifying the highest prevalence of asymptomatic *P. falciparum* in both the high and low parasite prevalence settings. However, parasite prevalence estimated by the HRP2 bead assay and PET-PCR had the highest level of inter-rater agreement relative to all the other tools tested and have the advantage of requiring fewer processing steps relative to Nested PCR and producing quantitative results.

**Supplementary Information:**

The online version contains supplementary material available at 10.1186/s12936-022-04078-w.

## Background

Asymptomatic parasite carriage in *Plasmodium falciparum* infections is a well-known phenomenon [1]. Previously, it was assumed that residents of high transmission areas were at a greater risk of harboring asymptomatic (subclinical) infections as a result of acquired immunity to clinical malaria developed over repeated exposures [1, 2]. However, recent studies conducted in low-transmission areas of malaria endemic countries, especially in Africa have identified a high prevalence of asymptomatic *P. falciparum* carriers [3]. Asymptomatic *Plasmodium* carriage in low transmission settings has been suggested to be responsible for 20–50% of all malaria transmission in those settings [4].

Recent estimates of high asymptomatic parasite carriage in low transmission settings could be due to the sensitivity of the parasite detection tools used, where highly sensitive molecular tools increase parasite prevalence estimates [5]. Light microscopy, the gold standard for laboratory confirmation of malaria [6] has a sensitivity of detection ranging from 30 to 50 parasites per microliter (p/µL) of blood [7] to 50–500 p/µL [6]. In addition to having low sensitivity, microscopy is dependent on the quality of reagents and the techniques used in preparing and staining the smear [8] as well as the expertise of the microscopist who examined the smear [9]. These limitations and the difficulty of deploying microscopy to all testing sites have led to the expansion of tools used in malaria diagnosis and detection of infection to include tools such as rapid diagnostic test (RDT) kits, with a sensitivity of ~ 100 p/µL [6, 9] and molecular tools such as polymerase chain reaction (PCR), with a sensitivity of about 2–5 p/µL of blood for Nested PCR [10] and 0.01 to 1 p/µL of blood for real- time PCR [11].

Although the main rationale to improve malaria diagnostic tools is to ensure prompt and accurate parasite detection and treatment of clinical cases, the new diagnostic tools are frequently used by Malaria Control Programmes to assess parasite carriage in population surveys [5, 12, 13].

Malaria RDT kits are predominantly based on the detection of *P. falciparum* histidine-rich protein (HRP2) and/or *Plasmodium* lactate dehydrogenase (LDH) antigens, and despite RDT kits having a similar sensitivity to microscopy [14–16], their ease of use and fast turnaround time have made them a preferred diagnostic tool [17, 18]. The most commonly used malaria RDT kits are the HRP2-based tests, because of the abundant production of the HRP2 protein by the parasite and its enhanced sensitivity compared to LDH based RDT kits [19, 20]. A major limitation of RDT kits is that they are not quantitative [21]. Additional limitations of HRP2 RDT kits include the persistence of HRP2 antigen in the blood for up to four weeks after the clearance of an active infection, which results in high false-positive rates [22] and the increasing reports of false-negative results due to the presence of parasites not producing HRP2 as a result of *pfhrp*2 gene deletions [23].

A recently developed tool for detecting parasite antigen is a sensitive HRP2 bead assay, which can simultaneously measure multiple parasite antigens including HRP2, LDH and aldolase. The HRP2 bead assay has a limit of detection of 0.24, 1.43 or 71.9 pg/mL for three unique forms of HRP2 antigens (Type A, B, and C, respectively) that are captured by the beads [24]. The main disadvantage of the HRP2 bead assay is that it cannot be used as a point of care test [24–26].

Molecular diagnosis of malaria largely comprises of the use of a wide variety of polymerase chain reaction (PCR) platforms to detect parasite nucleic acids. A photoelectron induced transfer PCR (PET-PCR), has a limit of detection of 3.2*,* 5.8, 3.5 and 5 p/µL for *P. falciparum*, *Plasmodium ovale*, *Plasmodium malariae* and *Plasmodium vivax,* respectively*,* and the possibility of multiplexing, which allows the detection of both *P. falciparum* and another human *Plasmodium* species in a single reaction [27]. PET-PCR has also been optimized for use in detecting asymptomatic malaria parasite carriers in large community surveys [24]. Although molecular tools are more sensitive than microscopy and RDTs, they are not suitable for point of care diagnosis as they are time-consuming and require expensive specialized equipment and reagents as well as highly-skilled personnel to run them [10].

This pilot study evaluated the utility of a variety of malaria parasite detection tools; microscopy, HRP2-based malaria RDT, HRP2 bead assay, PET-PCR and Nested PCR in determining the prevalence of asymptomatic *P. falciparum* parasite carriage amongst participants from two communities with varying malaria parasite prevalence in southern Ghana.

## Methods

### Ethical consideration

Ethical approval for the study was obtained from the Institutional Review Board of the Noguchi Memorial Institute for Medical Research (NMIMR), Ghana (Study number 089/14-15). Written informed consent, assent and parental consent (for children) were obtained from all study participants.

### Study site and population

This pilot study used consecutive sampling to select 194 participants from a larger cross-sectional study conducted in Obom and Asutusare during the off-peak malaria season (February 2016) [28]. Participants from the larger study were aged between 6 and 70 years old and selected based on the absence of any sign or symptom suggestive of malaria.

**Fig. 1:**
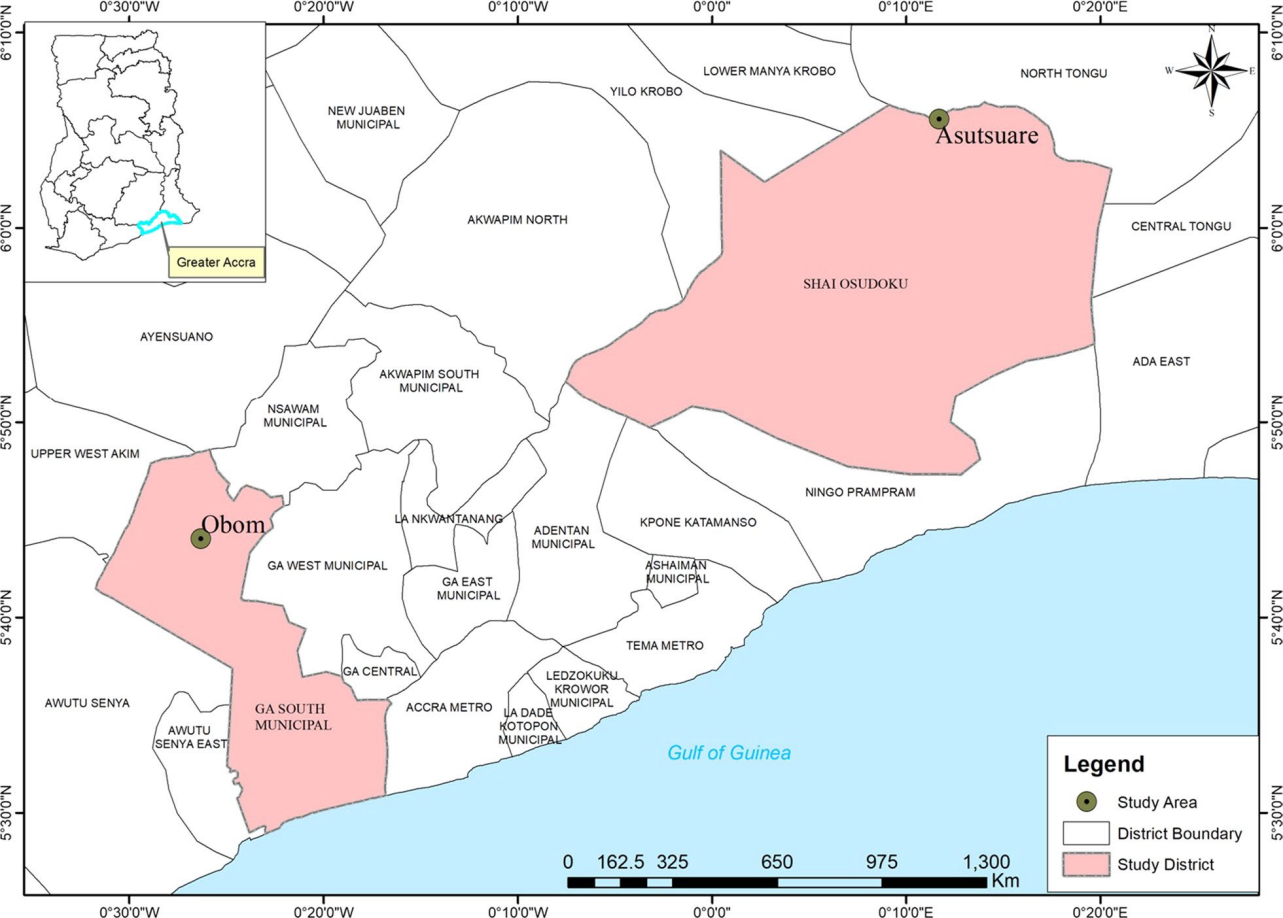
Map of Ghana highlighting the study sites. The study sites, Obom and Asutsuare are represented by green circles on the map. The map was created for this study by Awiah Dzantor Selorm, ACECoR, University of Cape Coast, using shapefiles from the Survey Department of the Ghana Statistical Services and ArcMap GIS v10.5

Obom is a high malaria parasite prevalence setting in the Ga South municipality of Greater Accra Region of Ghana (Fig. 1) with a microscopy estimated parasite prevalence of 35% in 2014 [5, 12] and 41.8% in 2019 [29]. Asutsuare is a low malaria parasite prevalence setting in the Shai Osudoku District of the Greater Accra Region of Ghana. Microscopy estimates of parasite prevalence in Asutsuare were 8.9% in 2009 [30] and 3.6% in 2016 [31]. According to the World Health Organization (WHO), an annual parasites prevalence of 1–10% is considered as low and ≥ 35% considered as high [32].

### Sample collection and processing

Before sample collection, the axillary temperature of each participant was measured using a digital thermometer. Venous blood (5 mL) was collected from each volunteer into EDTA vacutainer**®** blood collection tubes (BD, New Jersey, USA). An aliquot of the blood was used to prepare thick and thin blood smears for microscopy. The blood smears were air dried, fixed (thin- film only) and stained with Giemsa following the WHO standard protocol [8, 33]. The slides were observed at 100X magnification under a light microscope by two microscopists working independently. A sample was scored as negative for malaria if no parasite was seen after observing 200 fields and scored positive if parasites were observed. Parasite density, destimated as the number of parasites per microlitre blood (p/ μL) was determined as the number of malaria parasites observed per 200 white blood cells (WBCs) X 40, with the assumption that 1 μL of blood contains 8,000 WBCs [34].

Additionally, 5 μL of the blood was used for *P. falciparum* diagnosis using the Malaria Pf (HRP2) Ag RDT Multi Kit (Access Bio Inc, New Jersey, USA), following the manufacturer’s instructions.

Four, 50 μL drops of blood sample were spotted on Whatman #3 filter paper (GE Life sciences, USA). The filter paper blood spots were individually air dried and stored at room temperature in a sealed plastic bag containing a desiccant. The remaining blood from each volunteer was separated into plasma and packed blood cells, which were subsequently stored frozen at − 20 °C until required. All samples from the field were subsequently transported to the Immunology Department of the NMIMR, Ghana for further processing and analysis. An aliquot of the whole blood was sent to the Centers for Disease Control and Prevention (CDC, USA) for additional analysis.

### DNA extraction

DNA for the Nested PCR was extracted at the NMIMR from two 3 mm disks punched out of the DBS using the Chelex extraction method as previously described [35]. Whereas DNA for the PET-PCR was extracted at the CDC from 200 μL of packed blood cell pellets using the QIAamp DNA Mini Kits (Qiagen, USA) according to the manufacturer’s protocol. The DNA extracted from both procedures was either stored at 4 °C for immediate use or stored at − 20 °C for later use.

### Nested PCR

The Nested PCR amplification of the *P. falciparum 18S rRNA* gene was adapted from Singh et al. [36] with slight modification as previously reported [12]. Briefly, 200 nM dNTPs, 2 mM MgCl_2_, 133 nM each of forward (rPLU6) and reverse (rPLU5) primers (Additional file 1: Table S1) and 1 U OneTaq DNA polymerase (NEB, UK) was used to amplify the *18S rRNA* gene from 5 μL (~ 20 ng) of DNA in the primary PCR. The secondary PCR was performed using similar concentrations of reagents as in the primary reaction mix; however, rFal1 (forward) and rFal2 (reverse) primers were used to amplify 1 μL of the primary product. Genomic DNA from the 3D7 strain of *P. falciparum* (MRA 102G) was used as the positive control sample and distilled water (no template) served as the negative control sample. Positive and negative control samples were included in each PCR reaction set up. The amplified PCR products were separated alongside a 100 bp ladder (New England Biolabs, UK) on a 2% agarose gel stained with Ethidium bromide. The gels were subsequently viewed under ultra-violet light using the FUSION-FX7 advanced (Vilber Lourmat, Germany) chemiluminescence documentation system. All PCR assays were performed using the Eppendorf Mastercycler Nexus thermal cycler (Eppendorf, UK).

### PET-PCR

The multiplex PET-PCR assay was performed as previously described [27]. Briefly, the amplification of *Plasmodium* genus was performed in a 20 μL reaction containing 2 μL (~ 20 ng) of each DNA template, TaqMan Environmental buffer 2.0 (Applied BioSystems, USA), 125 nM each of forward and reverse primers (Additional file 1: Table S1) except for the *P. falciparum* HEX-labeled primer which was used at a 62.5 nM. The cycling parameters used were an initial denaturation at 95 °C for 10 min, followed by 45 cycles of denaturation at 95 °C for 10 s, annealing at 60 °C for 40 s and an extension at 72 °C for 30 s. Genomic DNA from the 3D7 strain of *P. falciparum* (CDC, USA) was used as a positive control. All assays were performed in duplicate and using the Agilent Mx3005pro thermal cycler (Agilent Technologies, USA).

### HRP2 bead assay

The HRP2 concentrations (pg/mL) of each sample was determined using an HRP2 bead assay previously described by Rogier et al. [24]. Briefly, a 6 mm disc was punched out of the dried blood spot (DBS) and incubated overnight in 200 μL of Buffer B (blocking buffer: 0.3% Tween 20, 0.5% bovine serum albumin, 0.1% casein, 0.5% polyvinyl alcohol, 0.5% polyvinylpyrrolidine, 0.05% NaN_3_, and 0.01% *Escherichia coli* extract diluted 20-fold). A total of 50 μL of each test sample, Buffer B (background) and negative control sample (pooled plasma from 86 US blood donors who tested negative for malaria antigen and IgG and whose individual HRP2 concentrations have previously been evaluated) were added in duplicate on each plate. Following the assay incubation steps, 100 μL PBS was added to each well and incubated at room temperature with shaking for 1 min. The plate was subsequently read on a Luminex-200 machine (Luminex Corporation, USA) with a target of 50 beads per reading.

### Data analysis

All samples that yielded visible fragments after agarose gel electrophoresis or CT values < 40 (the CT cut off for the PET-PCR was set at 40) after real time PCR analysis were classified as positive for the particular PCR reaction. The HRP2 antigen concentration in a sample was determined as the mean threshold fluorescence intensity (MFI) – the background signal obtained from reading the buffer (blank). The cutoff value for a positive sample was the lognormal mean of the average negative control MFI (obtained from 86 malaria naïve individuals) + 3 SD.

IBM SPSS version 20 was used to generate the descriptive statistics including median and to compare median age, haemoglobin and temperature between the two sites. Graph Pad Prism version 7 was used to determine Pearson Chi-Square for sex and parasite prevalence estimated by RDT, microscopy, Nested PCR and HRP2 bead assay, Mann–Whitney test for age and Cohen’s kappa test was used to determine the level of agreement between parasite prevalence estimates determined by two different tests (RDT, microscopy, Nested PCR and HRP2 bead assay). The Wilson-Brown diagnostic test was used to determine the diagnostic properties of the *Plasmodium* detection tools.

Statistical significance was set as P ≤ 0.05 unless otherwise stated. Kappa values of < 0 are classified as no agreement (disagreement), 0.0–0.20 are classified as poor agreement; 0.21 – 0.40 are classified as fair agreement; 0.41–0.60 are classified as moderate agreement and values of 0.61–0.80 classified as substantial agreement and 0.81–1.0 as an almost perfect agreement [37].

## Results

### Demographics

Of the 194 participants, 105 (54.1%) were residents of Obom, a high parasite prevalence area and 89 (45.9%) were residents of Asutsuare, a low parasite prevalence area. There was no significant difference (p = 0.652) in the distribution of males between the two study sites (53% in Asutsuare and 49% in Obom) (Table 1) or in terms of age (p = 0.109). The median (IQR) age of participants from Obom was 14 (12–24.3) years and the median (IQR) age in Asutsuare was 16 (13–25.8) years.

**Table 1:** Demographics of the study participants

Parameters	Obom (n = 105)	Asutsuare (n = 89)	P-value
Sex			
Male/Female	48/50*	43/38*	0.652^a^
Age (years)			
Median (Range)	14 (6.0–70.0)	16 (10.0–66.0)	0.109^b^
Diagnostics			
Microscopy	20/105 (19.1)	2/89 (2.2)	0.0002^a^
HRP2-RDT	39/101 (38.6)	–	–
HRP2 Bead (Luminex)	65/105 (61.9)	10/89 (11.2)	0.0001^c^
PET-PCR	63/105 (60)	5/89 (5.6)	0.001^c^
Nested PCR	70/98 (71.4)¥	42/83 (50.6)‡	0.0056^c^

*Yrs*  year, *Min* minimum, *Max* maximum, *n* = total number of samples tested.

^a^Pearson Chi-Square

^b^Mann Whitney (Two-tailed)

^c^Fisher’s exact test. *a few samples had missing gender data. ¥ = nPCR was not perform for 7 samples; ‡ = nPCR was not perform for 6 samples

### Estimation of parasite prevalence and density by microscopy

**Fig. 2:**
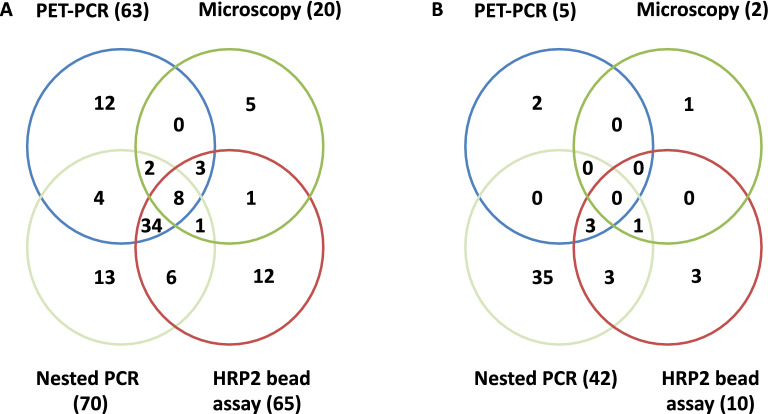
Comparisons of PET-PCR, nPCR and the HRP2 bead assay detection tools to Microscopy (Gold standard). **A** Venn diagram illustrating the number of positive parasites by the four methods, **A** High transmission site, the four methods identified 8 samples as positive for the parasites, 6 positive samples between Nested PCR and HRP2 bead assay, and 0 between Microscopy and HRP2 bead assay, and 4 positive samples between PET-PCR and N-PCR. **B** Low transmission, the four methods did not identified any samples as positive for the parasites, 3 positive samples between Nested PCR and HRP2 bead assay, and no positive parasite between Microscopy and HRP2 bead assay, and also no positive sample between PET-PCR and Nested PCR

**Fig. 3:**
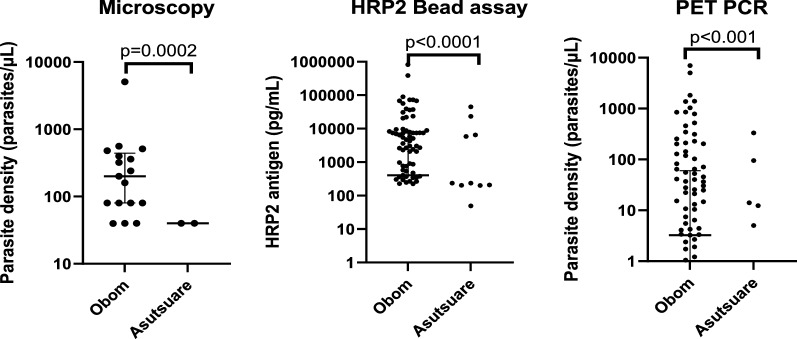
Parasite density determined by different tools. The median (IQR) parasite density of samples that tested positive for *P. falciparum* by microscopy (**a**), the median (IQR) HRP2 antigen content of the samples estimated using the HRP2 bead assay (**b**) and PET-PCR (**c**) from each site. Significant differences were observed in values obtained using microscopy (**a**) and the bead assay (**b**) but not by PET-PCR (**c**) when samples from Obom were compared to those from Asutusare

A total of 19.1% (20/105) and 2.2% (2/89) of the samples were identified as positive for *P. falciparum* by microscopy in the high (Obom) and low (Asutsuare) transmission sites respectively (Table 1; Fig. 2A, B). One of the samples from Obom contained a mixture of *P. falciparum* and *P. malariae* (however, this was not confirmed by PCR). A higher number of *P. falciparum* parasite carriers were detected in the high parasite prevalence setting (Obom) relative to the low parasite prevalence setting of (Asutsuare) (Pearson Chi-Square, p = 0.0002) (Table 1). Parasite density estimated as parasites per microlitre (p/µL) blood from Obom ranged between 32 p/µL and 5080 p/µL with a median (IQR) of 180 (80–405) p/µL, whilst in Asutsuare, both samples that tested positive by microscopy had a parasite density of 40 p/µL (Fig. 3A).

### Estimation of parasite prevalence based on antigen detection

The HRP2-RDT identified a total of 38.6% (39/101) of the samples collected from the high transmission area as positive. RDT results were not available for 4 samples from the high parasite prevalence area. None of the samples from the low parasite prevalence setting of Asutsuare tested positive by the HRP2 RDT (Fig. 2B; Table 1).

Detection of the *P. falciparum* HRP2 antigen using the HRP2 bead assay was significantly higher in Obom (61.9%) when compared to Asutsuare (11.2%), p < 0.0001. The *P. falciparum* HRP2 antigen levels of samples in Obom ranged from 226.0 to 820,368 pg/mL, with a median of 4689.0 and 49.4 pg/mL to 44,980 pg/mL with a median of 236.4 pg/mL in Asutsuare. The median HRP2 antigen levels in samples from Obom (4689.0 pg/mL) was significantly higher than samples from Asutsuare with median HRP2 antigen level of 236.4 pg/mL (Mann Whitney test, p < 0.0001) (Fig. 3B).

### Estimation of parasite prevalence based on molecular tests

In the high transmission setting (Obom), 60% (63/105) of the samples tested positive for *P. falciparum* by PET-PCR, with parasite density estimates ranging from 0.4 p/µL to 7,002 p/µL, with a median of 37.1 p/µL. In the low transmission setting (Asutsuare), 5.6% (5/89) of the samples tested positive for *P. falciparum*, with parasite density estimates ranging from 5.0 to 331.7 p/µL, with a median of 14.0 p/µL (Fig. 3C). Although a significantly higher number of parasites were detected in the high transmission setting (Obom) than in the low transmission setting (Asutsuare), (Fisher’s exact test, p < 0.001), there was no significant difference between the estimated parasite densities of the two sites when their median parasite density was compared (Mann Whitney test, p = 0.8879).

### Illustration of relationships among sensitive detection methods by areas

There were 8 and 12 samples from the high transmission setting (Obom) that tested positive and negative respectively for *P. falciparum* by all the five methods tested (Additional file 1: Fig. S3). In the low transmission setting, no sample was identified as positive by all the methods, whilst 38 samples were identified as negative by all five tests (Additional file 1: Fig. S1).

A total of 71.4% (45/63) of the PET-PCR positive samples and 28.5% (12/42) of the PET-PCR negative samples from the high transmission setting (Obom) tested positive by the HRP2 bead assay (Fig. 2, Additional file 1: Fig. S1). Whilst in Asutsuare, 80% (4/5) of the PET-PCR positive samples and 7.1% (6/84) of the PET-PCR negative samples tested positive by the HRP2 bead assay (Fig. 2, Additional file 1: Fig. S2).

### Comparison of detection tools

In the high transmission setting (Obom), parasite prevalence estimated by Nested PCR was significantly higher than that estimated by PET-PCR and the HRP2 bead assay (Pearson Chi square = 13.06 and 6.76, respectively, p < 0.001 for both), but parasite prevalence estimated by the HRP2 bead assay and PET-PCR were similar (Pearson Chi square = 31.89 and p > 0.05) (Fig. 2A, Additional file 1: Table S3).

In the low transmission setting, parasite prevalence estimated by the HRP2 bead assay was significantly higher than that recorded by PET-PCR (Fisher’s Exact Test p < 0.000) (Fig. 2B, Additional file 1: Table S1) and the difference between parasite prevalence estimated by both Nested PCR and PET-PCR on the one hand and Nested PCR and the HRP2 bead assay on the other were similar (Fisher’s Exact Test p = 1.000 and 0.156, respectively).

The HRP2 bead assay, identified a significantly higher number of *P. falciparum* positive samples compared to the HRP2 based RDT kit in the high malaria transmission setting (Pearson Chi-Square = 17.22, p < 0.001) (Table 1, Additional file 1: Table S3). Comparisons could not be made in the low transmission site, as no sample tested positive by HRP2 RDT (Table 1 and Additional file 1: Table S3). Nested PCR identified a significantly higher number of positive samples compared to PET-PCR in both the high transmission setting, Obom (Pearson Chi-Square = 13.06, p < 0.001) (Table 1, Fig. 2A) and the low transmission setting, Asutsuare (Fisher’s Exact Test, p < 0.001).

### Agreement between diagnostic tests

Microscopy is generally referred to as the gold standard diagnostic test for malaria. When results from the microscopy read out by the microscopists used in this study was set as the reference test (Table 2), the level of agreement between microscopy and the PET-PCR and the HRP2 bead assay tests in Obom was poor, with a fair agreement observed between results obtained by microscopy and RDT. In Asutsuare, the interrater agreement between microscopy and both PET-PCR and Nested PCR was poor but the agreement between microscopy and the HRP2 bead assay was fair. All the poor agreements were not significant, whilst the fair agreements were significant. There was no agreement between microscopy and Nested PCR in both Obom and Asutsuare (Table 2).

**Table 2:** Inter-rater agreement between different detection tools

Parameter	Obom Kappa (p value)	Asutsuare Kappa (p value)
RDT vs HRP2 bead assay	0.262 (0.004)*	
PET-PCR vs Nested PCR	0.348 (0.001)*	0.022 (0.665)
PET-PCR vs HRP2 bead assay	0.560 (0.000)	0.496 (0.000)
Microscopy vs RDT	0.194 (0.028)*	–
Microscopy vs PET-PCR	0.040 (0.537)	− 0.033 (0.727)
Microscopy vs HRP2 bead assay	0.027 (0.663)	0.134 (0.079)*
Microscopy vs Nested PCR	− 0.014 (0.808)	− 0.034 (0.768)
Nested PCR vs RDT	0.186 (0.019)*	–
Nested PCR vs PET-PCR	0.348 (0.001)*	0.022 (0.665)
Nested PCR vs HRP2 bead assay	0.248 (0.012)*	0.117 (0.084)
Nested PCR vs Microscopy	− 0.009 (0.874)	0.047 (0.157)

*Significant p value; vs, versus; No statistics could be computed for RDT vs the HRP2 bead assay in Asutsuare because no RDT positive samples were identified in Asutsuare

When Cohen’s kappa analysis (Table 2) was repeated with Nested PCR set as the reference, there was a poor agreement between Nested PCR and RDT but a fair agreement between Nested PCR and PET-PCR and the HRP2 bead assay in Obom, whilst in Asutsuare, all the agreements were poor. Excluding the microscopy data, all the agreements in Obom were significant whilst those in Asustuare were not significant (Table 2). In comparing diagnostic methods that measure similar parasite features, HRP2 antigen (RDT and the HRP2 bead assay) and parasite DNA (Nested PCR and PET-PCR), fair and significant agreements were observed only for the samples collected from the high transmission setting (Obom) (Table 2).

A crosstabulation analysis between PET-PCR and the HRP2 bead assay found that the two methods agreed moderately and significantly in Obom, Cohen kappa value = 0.560, p = 0.000 and in Asutsuare, Cohen kappa value = 0.496, p = 0.000) (Table 2).

### Sensitivity and specificity of diagnostic methods

The diagnostic properties of the three highly sensitive diagnostic tools were evaluated. In the high transmission setting (Obom), the diagnostic properties of Nested PCR, PET-PCR and the HRP2 bead assay were similar, whilst in the low transmission setting the diagnostic properties of only the HRP2 bead assay and PET-PCR were similar (Table 3). The sensitivity and specificity PET-PCR and the HRP2 bead assay at detecting asymptomatic *P. falciparum* carriage were similar in both the high (Obom) and low (Asutsuare) malaria transmission setting.

**Table 3:** Diagnostic properties of Nested PCR, PET-PCR and HRP2 bead assay

	Sensitivity (95% CI)	Specificity (95% CI)	PPV (95% CI)	NPV (95% CI)
Obom				
HRP2 bead assay vs Nested PCR	0.4815 (0.3989 to 0.5651)	0.4118 (0.3026 to 0.5304)	0.619 (0.5235 to 0.7062)	0.2857 (0.2057 to 0.3819)
HRP2 bead assay vs PET-PCR	0.5078 (0.4222 to 0.5929)	0.5122 (0.4059 to 0.6174)	0.619 (0.5235 to 0.7062)	0.4 (0.3114 to 0.4956)
Nested PCR vs PET-PCR	0.5263 (0.4419 to 0.6092)	0.6 (0.4829 to 0.7067)	0.7143 (0.6181 to 0.7943)	0.4 (0.3114 to 0.4956)
Asutsuare				
HRP2 bead assay vs Nested PCR	0.1923 (0.1080 to 0.3190)	0.3417 (0.2629 to 0.4303)	0.1124 (0.06219 to 0.1946)	0.494 (0.3891 to 0.5994)
HRP2 bead assay vs PET-PCR	0.6667 (0.4171 to 0.8482)	0.5153 (0.4391 to 0.5908)	0.1124 (0.06219 to 0.1946)	0.9438 (0.8751 to 0.9758)
Nested PCR vs PET-PCR	0.1064 (0.04630 to 0.2259)	0.328 (0.2519 to 0.4144)	0.05618 (0.02423 to 0.1249)	0.494 (0.3891 to 0.5994)

*PPV* Positive Predictive Value, *NPV* Negative Predictive value. The values reported are relative frequency with the 95% confidence interval (95% CI)

## Discussion

This study independently utilized five different diagnostic tools, PET-PCR, an HRP2 bead assay in addition to commonly used HRP2 RDT, microscopy and Nested PCR to determine the presence of *P. falciparum* harboured as asymptomatic infections in two communities with varied malaria parasite prevalence in southern Ghana. Asymptomatic malaria infections are usually characterized by low and submicroscopic parasite densities [38] and depending on the transmission intensity of the area, can contain lower than 100 parasites per microlitre (p/µL) [39, 40]. Relying solely on microscopy to detect the presence of *Plasmodium* parasites contained in such low density infections will likely result in missing many infections. Although microscopy, RDTs and Nested PCR are routinely used to detect malaria parasites in Ghana, PET-PCR and the HRP2 bead assay are known to be more sensitive than microscopy at detecting low density parasitaemia [24, 41] are rarely used. The sensitivities of various combinations of commonly used malaria diagnostic tools have been compared in different malaria endemic countries, including Ghana [5], none of the studies conducted in Ghana has compared the performance of PET-PCR and an HRP2 bead assay to microscopy, an HRP2-based RDT and Nested PCR at determining malaria parasite prevalence in different settings in Ghana. This study was conducted to evaluate the performance of malaria diagnostic tools, especially PET-PCR and the HRP2 bead assay as effective tools to detect asymptomatic malaria parasite carriage in settings with varying parasite prevalence in Ghana.

In this study, microscopy and HRP2-based RDT, the most commonly used malaria diagnostic tests in community surveillance studies in malaria endemic countries [42] produced the lowest estimates of asymptomatic parasite carriage in both the high and low malaria parasite prevalence settings. This was not surprising as the parasite densities of infections in samples from even the high parasite prevalence setting were very low. Asymptomatic infections are noted to contain low (submicroscopic) parasite densities [43], below the limit of detection of both microscopy and RDT kits [42].

The HRP2 RDT kit detected a higher number of samples as positive for *P. falciparum* than microscopy in the high transmission setting, but a reverse trend was observed in the low transmission setting. One likely reason for these results could be that the HRP2 antigen concentrations measured in samples from the high parasite prevalence setting were often higher and can be detected by the RDT than in the low parasite prevalence setting where it is below the detection limit of the RDT [51, 52]. Higher levels of HRP2 antigen could also result from a longer duration of antigen persistence in the high parasite prevalence setting due to more frequent infection. This would account for the higher positivity rates detected compared to microscopy in Obom but not in the low parasite prevalence setting (Asutsuare). The persistence of the HRP2 antigen after the clearance of infecting parasites is a well-known phenomenon [44, 45]. Consequently, HRP2 based malaria RDT kits may test positive for HRP2 antigens in the absence of an active infection. Additionally, as demonstrated in the study sites described here, parasite densities in low transmission settings are generally low and likely to be below the limit of detection of the RDT and microscopy especially in the off-peak season [31].

The diagnostic properties of PET-PCR, Nested PCR and the HRP2 bead assay was similar in the high parasite prevalence setting, and resulted in similar estimates of parasite carriage, however, the level of agreement among the three tests was low. This observation may be due to differences in limits of detection, assay targets and other fundamental differences between the methods. Persistence of HRP2 antigen for up to four weeks following a resolved *P. falciparum* infection can result in false positive HRP2 bead assay results, whilst parasites with deletions in the *Pfhrp*2 gene (not tested in this study) can cause false negative tests [46–48]. Nested PCR protocols generally have much higher numbers of amplification cycles compared to real time PCR protocols including PET-PCR and as such are likely to detect and amplify lower template concentrations than real time PCR. Nested PCR has previously been found to be more sensitive than PET PCR [49, 50]. However, the increased number of steps involved in Nested PCR make it more tedious and prone to contaminations and other operator errors that can increase the number of false negative as well as false positive test results compared with real time PCR processes.

When the results obtained from PET-PCR and Nested PCR, both DNA-detecting tools were compared to the results from the HRP2 bead assay, there was a much higher level of agreement between PET-PCR and the HRP2 bead assay. A possible explanation for this could be that PET-PCR and the HRP2 bead assay have a similar parasitaemia threshold of approximately two parasites per microliter [24], which is higher than that of Nested PCR. However, both the HRP2 bead assay and PET-PCR are quantitative, require fewer processing steps, and are faster processes than Nested PCR.

## Limitations

This pilot study was not formally designed as a diagnostic study. The different diagnostic tests used in this study detect different parasite components and also have varying limits of detection. The samples used in this study were collected during the off-peak malaria season where parasite densities are generally low and thus would require diagnostic tests with a low limit of detection and high sensitivity to detect. Also, deletions in the *Pfhrp*2 gene, which were not determined in the study could affect the sensitivity of both the HRP2 RDT and the HRP2 bead assay results.

## Conclusion

Nested PCR exhibited the highest sensitivity by identifying the highest prevalence of asymptomatic *P. falciparum* in both the high and low parasite prevalence settings. However, parasite prevalence estimated by the HRP2 bead assay and PET-PCR had the highest level of inter-rater agreement relative to all the other tools tested and have the advantage of requiring fewer processing steps and producing quantitative results relative to Nested PCR. These advantages make PET-PCR and the HRP2 bead assay very useful tools for detecting and estimating malaria parasite density especially amongst asymptomatic individuals during community surveys.

## Supplementary Information

**Additional file 1: Table S1**. Primer properties. **Table S2**. Summary of parasite prevalence data. **Table S3**. Comparison between the three sensitive methods in the two study sites. **Figure S1**. Illustrative Flow chart showing the total number of positive and negative samples detected by the combination of 5 different of *P. falciparum* diagnosis tools in the high and low malaria transmission setting. **Figure S2**. Illustrative Flow chart showing asymptomatic malaria diagnosis by the three sensitive diagnostic tools in the high transmission setting. **Figure S3**. Illustrative Flow chart showing asymptomatic malaria diagnosis by the three sensitive diagnostic tools in the low transmission setting.

## Data Availability

All data generated or analysed during this study are included in this published article.

## Abbreviations

RDT: Rapid diagnostic test
PET-PCR: Photo-induced electron transfer polymerase chain reaction
WBCs: White blood cells
PD: Parasite density
p/µL: Parasites per microlitre
