# Supplementary material for: Detecting asymptomatic carriage of Plasmodium falciparum in southern Ghana: utility of molecular and serological diagnostic tools
Source: Malar J. 2022 Feb 19;21:57. doi: 10.1186/s12936-022-04078-w (PMC8858553; doi:10.1186/s12936-022-04078-w)
Supplement: Supplementary file 1 — Additional file 1: Table S1. Primer properties. Table S2. Summary of parasite prevalence data. Table S3. Comparison between the three sensitive methods in the two study sites. Figure S1. Illustrative Flow chart showing the total number of positive and negative samples detected by the combination of 5 different of P. falciparum diagnosis tools in the high and low malaria transmission setting. Figure S2. Illustrative Flow chart showing asymptomatic malaria diagnosis by the three sensitive diagnostic tools in the high transmission setting. Figure S3. Illustrative Flow chart showing asymptomatic malaria diagnosis by the three sensitive diagnostic tools in the low transmission setting. [file 12936_2022_4078_MOESM1_ESM.docx]

**Table S1** Primer properties

|  | Name | Sequence | Annealing Temp |
| --- | --- | --- | --- |
| N-PCR | | | |
| Nest 1 | rPLU6 | TTA AAA TTG TTG CAG TTA AAA CG | 55 °C |
|  | rPLU5 | CCT GTT GTT GCC TTA AAC TTC | 55 °C |
| Nest 2 | rFAL1 | TTA AAC TGG TTT GGG AAA ACC AAA TAT ATT | 58 °C |
|  | rFAL2 | ACA CAA TGA ACT CAA TCA TGA CTA CCC GTC | 58 °C |
| PET-PCR |  |  |  |
| *Plasmodium* | 18s For | GGC CTA ACA TGG CTA TGA CG | 63 °C |
| *Plasmodium* | 18s Rev (FAM-labeled) | AGG CGC ATA GCG CCT GGC TGC CTT CCT TAG ATG TGG TAG CT | 63 °C |
| *P. falciparum* | For | ACC CCT CGC CTG GTG TTT TT | 63 °C |
| *P. falciparum* | Rev (HEX-labeled) | AGG CGG ATA CCG CCT GGT CGG GCC CCA AAA ATA GGA A | 63 °C |

*N-PCR, nested 18S rRNA gene PCR*

Table S2. Summary of parasite prevalence data

| Site | Microscopy | | PET-PCR | | N-PCR | | RDT | | Luminex | |
| --- | --- | --- | --- | --- | --- | --- | --- | --- | --- | --- |
|  | Pos | Neg | Pos | Neg | Pos | Neg | Pos | Neg | Pos | Neg |
| Obom | 20 | 85 | 63 | 42 | 70 | 28 | 37 | 61 | 65 | 40 |
| Asutsuare | 2 | 87 | 5 | 83 | 42 | 41 | 0 | 81 | 10 | 79 |
| Total | 22 | 172 | 68 | 125 | 112 | 69 | 37 | 142 | 75 | 119 |

Pos, positive; Neg, negative; N-PCR, nested PCR

Table S3. Comparison between the three sensitive methods in the two study sites.

| Site | Methods | Chi square value | p-value |
| --- | --- | --- | --- |
| Obom | Nested-PCR vs PET-PCR | 13.06 | <0.0001 |
|  | Nested-PCR vs Luminex | 6.76 | 0.009 |
|  | Luminex vs PET-PCR | 31.89 | 0.000 |
| Asutsure |  | Fisher’s Exact Test |  |
|  | Nested-PCR vs PET-PCR |  | <0.0001 |
|  | Nested-PCR vs Luminex |  | 0.156 |
|  | Luminex vs PET-PCR |  | 0.000 |

*PET-PCR, Photo-induced Electron Transfer Polymerase Chain Reaction; Luminex, Bead-based Assay; vs, versus.

**Figure S1.** Illustrative Flow chart showing the total number of positive and negative samples detected by the combination of 5 different of *P. falciparum* diagnosis tools in the high and low malaria transmission setting.

Pos, positive; neg, negative; micro, microscopy; Luminex, HRP2 bead assay, N-PCR, Nested PCR

**Figure S2.** Illustrative Flow chart showing asymptomatic malaria diagnosis by the three sensitive diagnostic tools in the high transmission setting.

Bead assay, HRP2 bead assay; PET-PCR, Photo-induced Electron Transfer Polymerase Chain Reaction; N-PCR, Nested Polymerase Chain Reaction; pos, Positive and neg, Negative. Seven samples had missing Nested PCR data

**Figure S3.** Illustrative Flow chart showing asymptomatic malaria diagnosis by the three sensitive diagnostic tools in the low transmission setting.

Bead assay, HRP2 bead assay; PET-PCR, Photo-induced Electron Transfer Polymerase Chain Reaction; N-PCR, Nested Polymerase Chain Reaction; pos, Positive and neg, Negative. Six samples had missing Nested PCR data.
